# Study on thermal conductivity of improved soil under different freezing temperatures

**DOI:** 10.1371/journal.pone.0292560

**Published:** 2023-10-18

**Authors:** Hongqi Wang, Dongwei Li, Minghai Xia, Xiufei Li

**Affiliations:** 1 School of Civil and Architectural Engineering, East China University of Technology, NanChang, China; 2 School of Architectural Engineering, Dalian University, Dalian, China; 3 Irrigation Management Department of Water Conservancy Engineering in Kuitun River Basin, Kuitun, Ili Kazakh Autonomous Prefecture, China; 4 China Nuclear Huatai Construction Co., Ltd., Shenzhen, China; Mirpur University of Science and Technology, PAKISTAN

## Abstract

Based on the influence of moisture content, dry density and temperature (≦ 0°C) on the thermal conductivity of lime-modified red clay, the thermal conductivity was measured by transient hot wire method. A total of 125 data were obtained and the evolution law of thermal conductivity with influencing factors was analyzed. The fitting formula of thermal conductivity of lime-modified red clay and a variety of intelligent prediction models were established and compared with previous empirical formulas. The results show that the thermal conductivity of lime-modified red clay increases linearly with water content and dry density. The change of thermal conductivity with temperature is divided into three stages. In the first stage, the thermal conductivity increases slowly with the decrease of temperature in the temperature range of-2°Cto 0°C. In the second stage, in the temperature range of-5°Cto (-2)°C, the thermal conductivity increases rapidly with the decrease of temperature. In the third stage, in the range of-10°Cto (-5)°C, the thermal conductivity changes little with the decrease of temperature, and the fitting curve tends to be stable. The fitting formula model and various intelligent prediction models can realize the accurate prediction of the thermal conductivity of lime-improved soil. Using RMSE (Root Mean Square Error) and MAPE (Mean Absolute Percentage Error) to evaluate the model, it is found that the GBDT decision tree model has the best prediction effect, the RMSE value of the predicted value is 0.084, and the MAPE value is 4.1%. The previous empirical models have poor prediction effect on the thermal conductivity of improved red clay. The intelligent prediction models such as GBDT decision tree with strong universality and high prediction accuracy are recommended to predict the thermal conductivity of soil.

## 1. Introduction

Red clay is a special soil with strong water sensitivity and easy agglomeration. It is often improved by lime, cement and other materials. Many scholars have made many research results on the stress field of lime and cement improved red clay, but few on the temperature field of improved soil.

Thermal conductivity is an important parameter to study the soil temperature field, which determines the distribution of soil temperature field [1]. Numerous studies have been conducted based on the effects of saturation, porosity, dry density, and mineral composition on the thermal conductivity of soils.

Yunshan X, Jie H, Jishi G et al. [2–4] studied the evolution of clay thermal conductivity under the influence of high temperature, water content and other factors through laboratory tests. The influence mechanism of high temperature and other factors on soil thermal conductivity was analyzed. Zhipeng L et al. [5] studied the influence of biochar content and water content on the thermal conductivity of red clay by thermal pulse method, and analyzed the promotion mechanism of biochar on improving the thermal conductivity of soil. Lei Z and Balaji C N et al. [6, 7] studied the influence of different cement, temperature and moisture content on the thermal conductivity of soil. The evolution of thermal conductivity with cement content, water content and temperature is summarized.Yunshan X et al. [8] studied the effect of dry-wet cycle on the thermal coefficient of red clay by thermal probe method. Combined with microscopic mechanism, the evolution mechanism of thermal conductivity of red clay under dry and wet conditions was explained. Xiangtian X [9] studied the variation of soil thermal conductivity in a certain range of temperature, moisture content and dry density. The results show that the thermal conductivity decreases linearly with temperature in the positive temperature range, and increases exponentially with temperature in the negative temperature range. The prediction effect of Johansen model and CK model was evaluated by linear regression analysis. Orakoglu M E et al. [10] studied the influence of different fiber content, temperature and freeze-thaw cycles on the thermal conductivity of soil by using the transient hot wire thermal conductivity instrument. The evolution law of thermal conductivity was analyzed. The results show that the thermal conductivity of fiber reinforced soil is negatively correlated with temperature and freeze-thaw cycles, and the thermal conductivity of all steel fiber and 1% basalt fiber reinforced soil decreases significantly. The physical statistical model is established, which provides a reference for the study of soil thermal conductivity. Kaiqi L [11] explained the random distribution of voids by establishing a finite element model. The effective thermal conductivity of frozen-half frozen-unfrozen soil was calculated. The prediction effect of the numerical method on the thermal conductivity considering the influence of soil, porosity and saturation is verified. Chenyang L et al [12] studied the effects of moisture content, porosity and density on the thermal conductivity of undisturbed soil. Through weight analysis, the results show that the influence weights of natural density, moisture content and porosity on thermal conductivity are 30.98%, 55.57% and 13.45%, respectively. A variety of empirical models and BP neural network model are compared to achieve accurate prediction of soil thermal conductivity. Tao Z et al. [13] established an artificial neural network prediction model for soil thermal conductivity based on soil thermal conductivity mechanism. The results show that all models have achieved high accuracy, and the average absolute error and root mean square error are less than 0.360 and 1.000 W·m^-1^·K^-1^, respectively. The generalized artificial neural network model has the best prediction effect and the highest accuracy. Kaiqi L et al. [14] predicted the thermal conductivity of soil by establishing a variety of intelligent prediction models. Based on the correlation analysis, the weight of each influencing factor is calculated. The results show that among the six machine learning algorithms, the adaptive enhancement model has the best prediction effect, with the minimum error RMSE = 0.099 and R^2^ > 0.98. Based on the database of soil thermal conductivity, Caijin W et al. [15] established an artificial neural network prediction model and Monte Carlo simulation, and obtained a good prediction effect, and realized the intelligent prediction of thermal conductivity. Kardani N et al. [16] proposed a new soil thermal conductivity prediction model by integrating artificial neural network (ANN) and particle swarm optimization (POS) optimization and adaptive methods, including ANN-IPOS (ANN optimized by improved POS) and ANN-APOS (ANN optimized by improved APOS). The results show that the proposed ANN-APOS model has the best prediction performance and can be used to predict the thermal conductivity of unsaturated soil and other engineering problems. Caijin W et al. [17] studied the influence of various factors on soil thermal conductivity by establishing intelligent prediction models such as artificial neural network (ANN) and support vector machine (SVM). The results showed that the SVM model was the best, and all intelligent prediction models were superior to the traditional empirical models. Yizheng D et al. [18] used clustering algorithm to study the relationship between thermal conductivity and dry density during soil drying. The results showed that the overall performance of the model was the best with 1.4 g·cm^-3^ as the demarcation point and different algorithms. The new model is simpler and more applicable than the previous models, and is suitable for studying the thermal conductivity of dry soil under different dry densities.

At present, intelligent prediction models are widely used in various fields due to their high accuracy, fast calculation speed and strong learning ability. Many scholars have established intelligent prediction models through machine learning, such as BP neural network, decision tree and linear regression models to carry out research on prediction work. However, in the study of thermal conductivity of soil, many scholars mainly predict the thermal conductivity of plain soil. In the modeling and prediction of thermal conductivity of improved soil, it is rare. At the same time, there is still a gap in the research on the prediction and comparison of improved soil by establishing a variety of intelligent prediction models.

In summary, the research on thermal conductivity of clay is mainly focused on the study of plain clay. In the study of thermal conductivity of improved clay, the change law of lime improved red clay under the influence of low temperature and other factors still needs to be further studied. Regarding the prediction models, the traditional prediction models are more complicated. The empirical model has low universality. Intelligent prediction models are less applied to the prediction of thermal conductivity of improved soils. Therefore, it is important to study the evolution law of lime improved red clay soil under the influence of factors such as moisture content, dry density and temperature (≦0°C), and to establish the analysis of fitting equation model and multiple intelligent prediction models for comparison, which is important for future research on thermal conductivity and temperature field of the same soil samples and their improved soils.

## 2. Materials and methods

### 2.1 Materials

The red clay used in the test was taken from the side of Nanchang construction site. Basic physical parameters are shown in Table 1.

**Table 1:** Basic physical parameters of red clay.

Soil sample	Optimal water content(%)	Maximum dry density(g·cm^−3^)	Plastic limit(%)	Liquid limit(%)	Specific gravity
Red clay	21.33	1.66	17.26	45.99	2.74

Lime is lime with 99% calcium content and particle size less than 0.5 mm.

### 2.2 Test scheme

Based on previous studies, when the lime content is 5%-7% [19–21], the mechanical properties of the improved soil are the best. Therefore, the fixed lime content is 6%. Based on the minimum temperature of-10°C in winter in Dalian area, the temperature gradient is set up. The specific test scheme is shown in Table 2.

**Table 2:** Test scheme.

moisture content(%)	Dry density(g·cm^−3^)	Temperature(°C)	lime content(%)
19%、21%、23%、25%、27%	1.2、1.3、1.4、1.5、1.6	0、-3、-5、-7、-10	6%

### 2.3 Test instrument and processes

The test instrument adopts Xiaxi TC3000E transient hot wire method thermal conductivity instrument and a set of self-matching circulating cold bath system. The test procedure is as follows:(i)Red clay with particle size less than 0.5mm was selected and dried at 105°C;(ii)according to the test plan to soil samples and stand for more than 12 h; (iii)Φ61.8 * 20 mm, 60 cm^3^ sample mold, pressed into standard sample;(iv)The sample mold with a specification of Φ61.8 * 20 mm, 60 cm^3^ was pressed into a standard sample, wrapped with a preservative film and stored in a curing box for more than 6 h.(v)Control the thermal conductivity measurement environment box temperature and sample temperature to the target value, then the thermal conductivity measurement.

## 3. Results

### 3.1 Effect of moisture content on thermal conductivity

**Fig 1:**
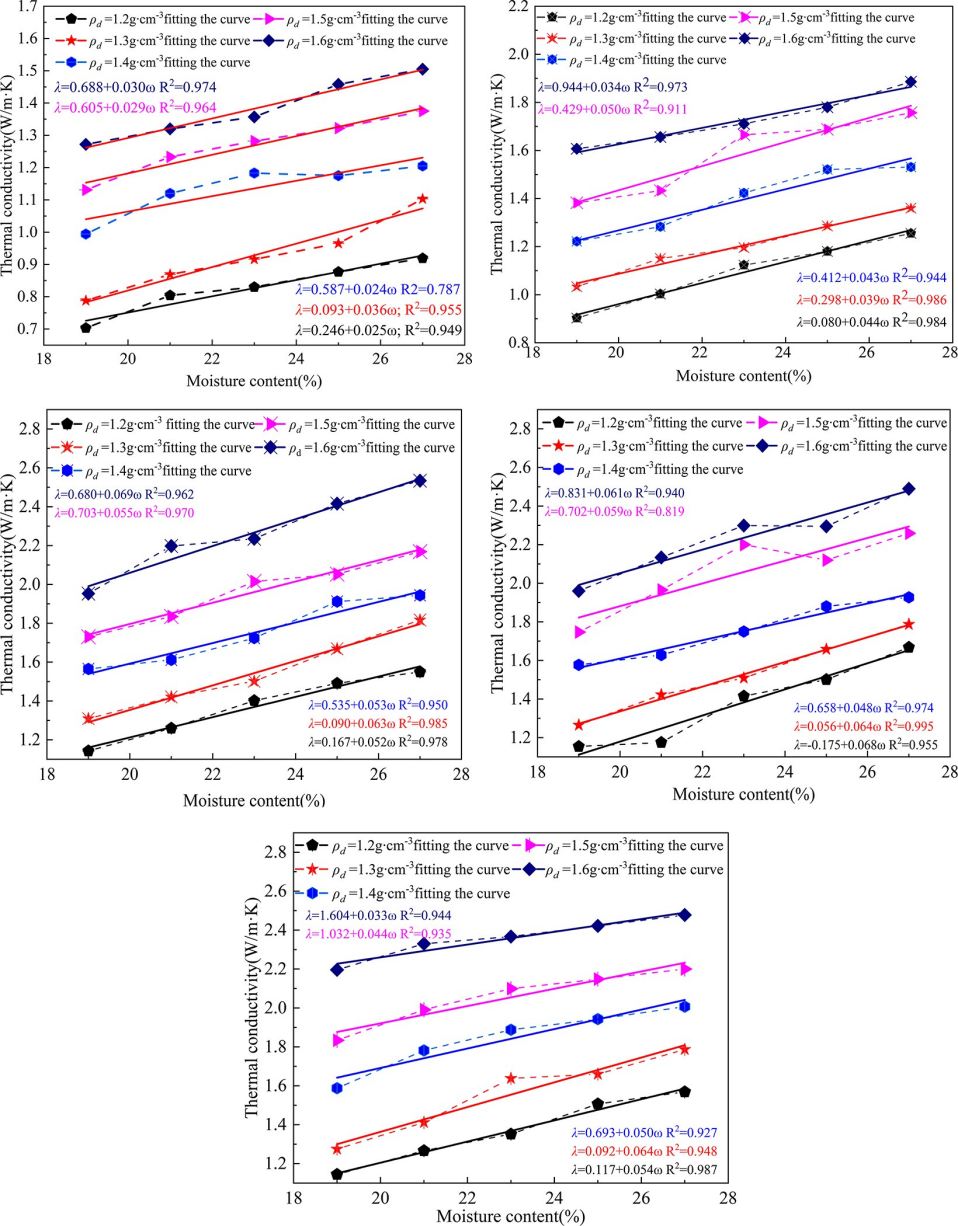
Soil thermal conductivity versus moisture content.

The thermal conductivity of lime modified red clay permafrost varies with moisture content in the range of different temperatures and dry densities as shown in Fig 1. As can be seen from the line drawing of the original data points in the Fig, the thermal conductivity of frozen soil gradually increases with the increase of moisture content. Through the linear fitting of the imaginary line of the original data in the graph, it is found that the overall accuracy of the fitting curve is high.

The permafrost thermal conductivity increases linearly with the increase of moisture content. The permafrost thermal conductivity increases with moisture content by 0.244, 0.328, 0.463, 0.486, and 0.400 W·m^-1^·K^-1^on average at different temperatures, respectively. The thermal conductivity increases by an average of 0.048 W·m^-1^·K^-1^ for every 1% increase in moisture content, in the range of 19–27% moisture content.

The volume of water (or ice) inside the permafrost increases, the volume of air decreases, *λ*_*ice*_ *> λ*_*water*_ *> λ*_*air*_, and the thermal conductivity of the soil increases as the moisture content increases.

### 3.2 Effect of dry density on thermal conductivity

**Fig 2:**
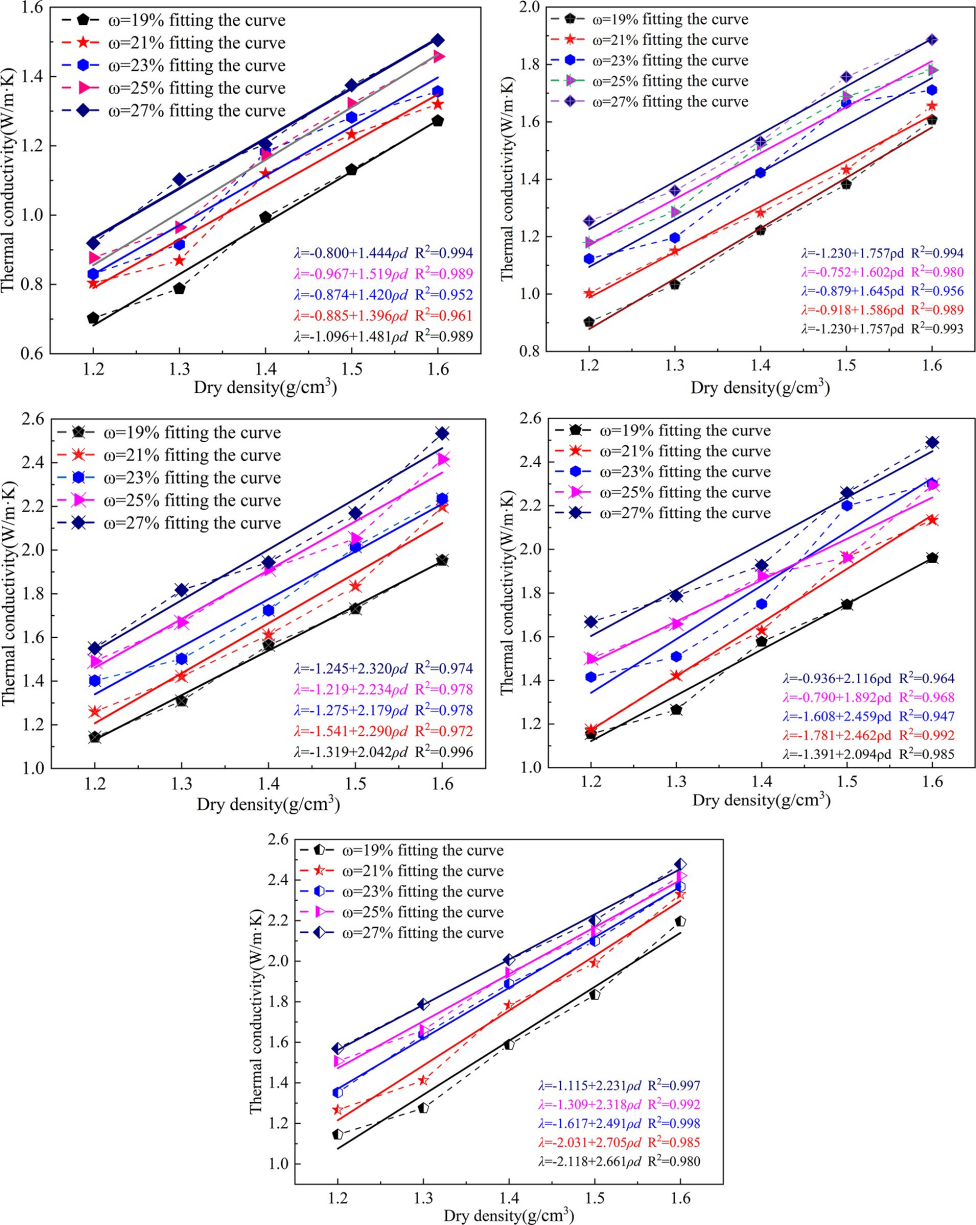
Variation of thermal conductivity of soil with dry density.

The relationship between the thermal conductivity of the improved soil and the dry density under different temperature and moisture content conditions, as shown in Fig 2. As can be seen from the original data points in the Fig, the thermal conductivity of frozen soil increases with the increase of dry density. By fitting the original data points of thermal conductivity of frozen soil with dry density, it can be seen that the thermal conductivity of frozen soil has a linear relationship with dry density. It can be seen from the fitting curve that the overall fitting accuracy is high.

The thermal conductivity of permafrost all increases linearly with dry density. The thermal conductivity index of the improved soils at different temperatures increased on average with the overall dry density by 0.556, 0.635, 0.898, 0.853, and 0.991W·m^-1^·K^-1^, respectively, in the range of moisture content.

The dry density gradually increases, the contact between soil particles becomes closer, the porosity decreases and the pore space decreases. Since the heat transfer properties of soil particles are higher than those of air, the thermal conductivity of the soil gradually increases with the increase in dry density.

### 3.3 Effect of temperature on thermal conductivity

**Fig 3:**
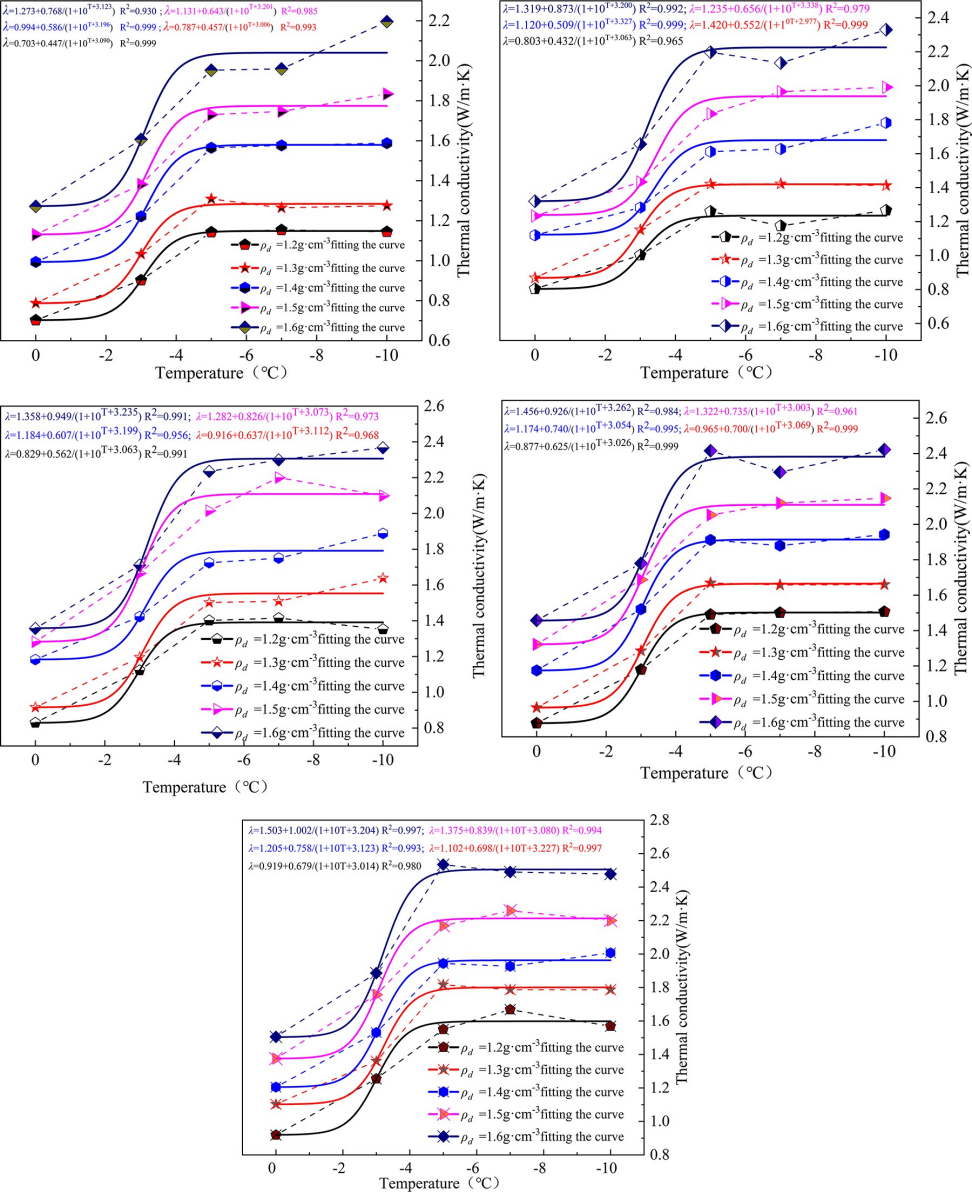
Soil thermal conductivity as a function of temperature.

The relationship between thermal conductivity and temperature in the temperature range of -10°C to 0°C for soils with different moisture contents is shown in Fig 3 As can be seen from the dotted lines in the Fig, with the change of temperature, the thermal conductivity of frozen soil increases at different rates with temperature, and the change of thermal conductivity of frozen soil varies greatly in different temperature ranges. Through the curve fitting of the original data points of the thermal conductivity of frozen soil, the change trend conforms to the power function relationship, and the fitting accuracy is high. It shows that the curve model can be used to characterize the variation of thermal conductivity of soil with temperature and other influencing factors.

As shown in the fitted curve in Fig 3, the thermal conductivity of the improved soil decreases with temperature under the same moisture content and dry density conditions, and the overall change of thermal conductivity is divided into three stages. In the first stage, the thermal conductivity rises slowly with decreasing temperature in the temperature interval of -2°C~0°C. The overall variation in thermal conductivity is small. In the second stage, the thermal conductivity shows a rapid increase with decreasing temperature in the temperature interval of -5°C to (-2)°C. In the third stage, the thermal conductivity changes less with decreasing temperature in the interval of -10°C to (-5)°C. The temperature decreases from-5°C to (-10)°C, and the fitting results were approximately stabilized.

### 3.4 Analysis

Moisture content is one of the main influencing factors of the thermal conductivity of permafrost. As the moisture content increases, the solid-gas heat transfer volume of the soil decreases, the inter-solid heat transfer volume increases, and the thermal conductivity of the soil increases.

With the increase of dry density, the closer the contact between particles, the porosity of the soil gradually decreases, the heat transfer area between particles increases, the heat transfer performance is enhanced, and the thermal conductivity of the soil increases.

Pure water freezes into ice, which is the temperature node of ice-water phase transition at 0°C. Soil contains more minerals and impurities. The freezing temperature of water in soil is lower than that of pure water. As can be seen from Fig 3, the thermal conductivity changes less in the range of -2°C to 0°C under the improved soil as the temperature decreases and a small amount of water inside the soil freezes into ice. When the temperature was below -2°C, the thermal conductivity of the improved soil changed rapidly with the decrease of temperature. In the interval of -5°C to (-2)°C, the amount of water inside the soil freezes gradually with the decrease of temperature, and the thermal conductivity of the soil increases rapidly. When the temperature reaches -5°C, the unfrozen moisture content inside the soil is less. As the temperature decreases, the unfrozen moisture content freezes into ice gradually decreases. When the temperature is lower than -5°C, the thermal conductivity of the improved soil changes less as the temperature decreases. The fitted curve shows that the thermal conductivity basically tends to be stable.

## 4. Predictive models

At present, in the thermal conductivity prediction model, there are mainly: theoretical model, empirical model and intelligent prediction model. The intelligent prediction model mainly uses machine learning to establish a variety of models, such as artificial neural network, decision tree, linear regression, support vector machine and so on.

Based on the influence of moisture content, dry density, temperature and other factors on the thermal conductivity of lime-modified red clay, 125 data were tested and collected. Based on all the data, the empirical formula and intelligent prediction model are established. The intelligent prediction models include: BP neural network model, convolutional neural network model, radial basis neural network model, support vector machine model, and decision tree model. The support vector machine model includes: linear kernel, Gaussian kernel and polynomial kernel function algorithm. The decision tree model includes: conventional decision tree model, GBDR decision tree and forward incentive decision tree. By comparing the empirical model, the intelligent prediction model and the previous empirical model, the best prediction model is provided for the study of the thermal conductivity of lime-modified red clay.

### 4.1 Empirical models

Two empirical formulas for predicting the thermal conductivity of permafrost under the influence of temperature have been proposed in the literature [22] as follows:

$$\lambda =(24.25\rho_{d}‐9.83\rho_{d}^{2}‐15.81)\;\omega T+(4.75\rho_{d}-2.44)\omega$$ (1)

$$\lambda =(-0.0587T+1.034)(\rho_{d}-0.7)(1.083+0.0706S_{r}+0.0241S_{r}^{2})$$ (2)

Where *λ* represents the thermal conductivity of the soil in W·m^-1^·K^-1^; *ρ*_*d*_ represents the dry density of soil in g·cm^-3^; ω represents the moisture content in %; T represents the temperature in °C; *S*_*r*_ represents saturation in %.

**Fig 4:**
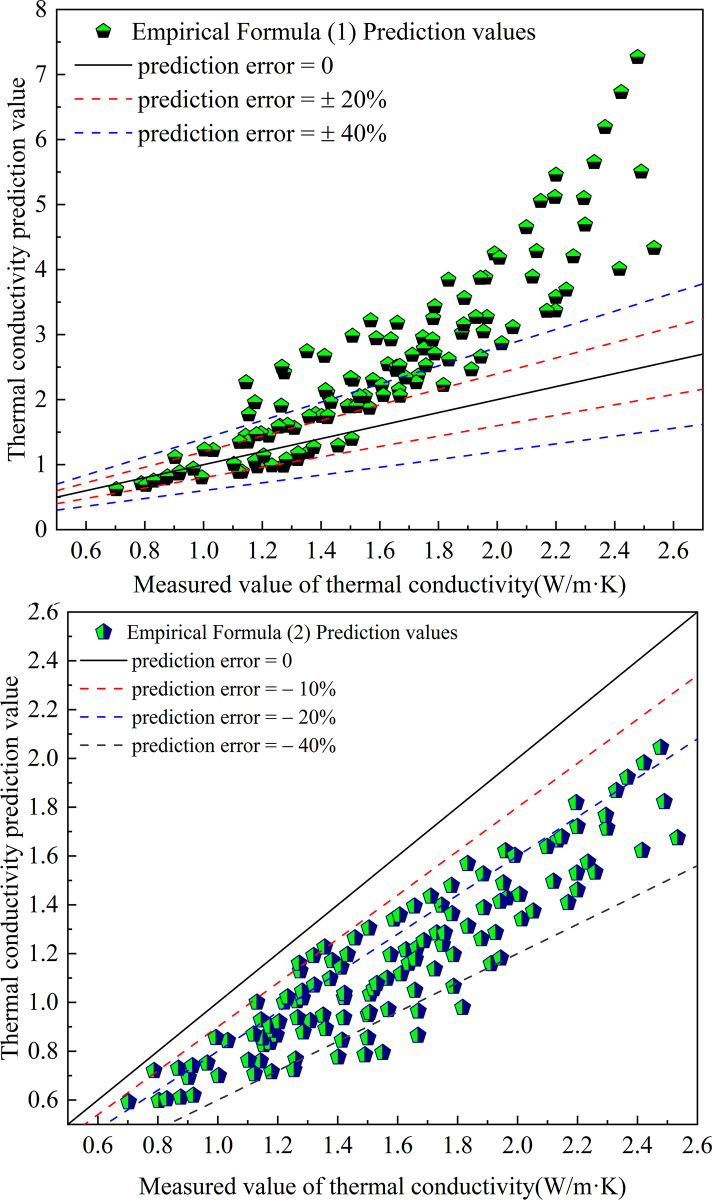
Prediction results of previous empirical models.

The thermal conductivity of the improved soil was predicted by the previous empirical model, and the results are shown in Fig 4.

The empirical model Eqs (1) and (2) predicted 48.8% and 92% of the predicted values with errors less than 40%, respectively. 21.6% and 28% of the predicted values, respectively, with an error of less than 20%. The mean square errors of the predicted values of the two formula models are 1.8 and 0.216, respectively.

The two empirical formulas have large prediction error of thermal conductivity of lime-modified red clay under the influence of moisture content, dry density and temperature, which is not universal.

Based on the effects of moisture content, dry density and temperature on the thermal conductivity of improved soils, the following empirical equation model was developed.

$$\lambda =A_{2}+\frac{A_{1}-A_{2}}{1+10^{T-A_{3}}}$$ (3)

Where *λ* is the thermal conductivity of soil in: W·m^-1^·K^-1^; *A*_*1*_, *A*_*2*_, *A*_*3*_ are fitting parameters, affected by moisture content and dry density; T is the temperature (-10°C to 0°C);

Combined with Fig 3.1 and 3.2, the relationship between *A*_*1*_, *A*_*2*_ and moisture content, dry density is established as follows.

$$A_{1}=-2.786+0.058\omega +2.309\rho_{d}$$ (4)

$$A_{2}=-1.589+0.029\omega +1.453\rho_{d}$$ (5)

$$A_{3}=-2.548+0.412\rho_{d}$$ (6)

where ω is the moisture content in % and *ρ*_*d*_ is the dry density in g·cm^-3^.

Bringing Eqs (4) to (6) into Eq (3) yields the new fitted empirical formula, as shown in Eq (7).

$$\lambda =-1.589+0.029\omega +1.453\rho_{d}+\frac{-1.197+0.029\omega +0.856\rho_{d}}{1+10^{T-(0.412\rho_{d}-2.548)}}$$ (7)

The meaning of each parameter in the formula is the same as above.

**Fig 5:**
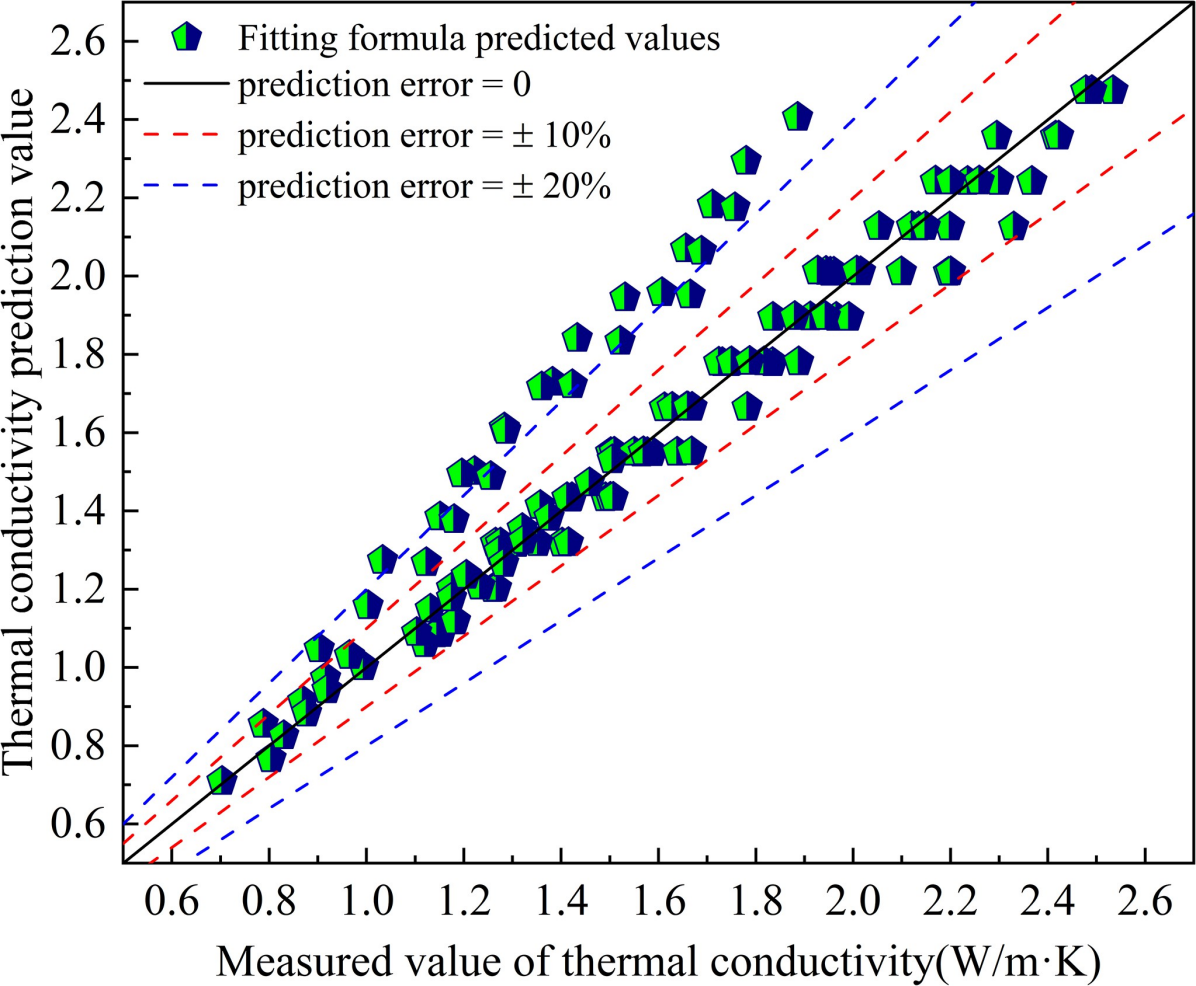
Predicted thermal conductivity of the fitted equation.

The prediction results of the fitted empirical formula are shown in Fig 5. It was found that the thermal conductivity of improved soils was predicted using the fitted empirical formula. 80% of the predicted values had an error of less than 10%. The maximum prediction error was 28.9% and the minimum prediction error was 0. The mean square error of the predicted values was 0.026. The accurate prediction of thermal conductivity of improved soils was achieved.

### 4.2 Multiple intelligent prediction models

**Fig 6:**
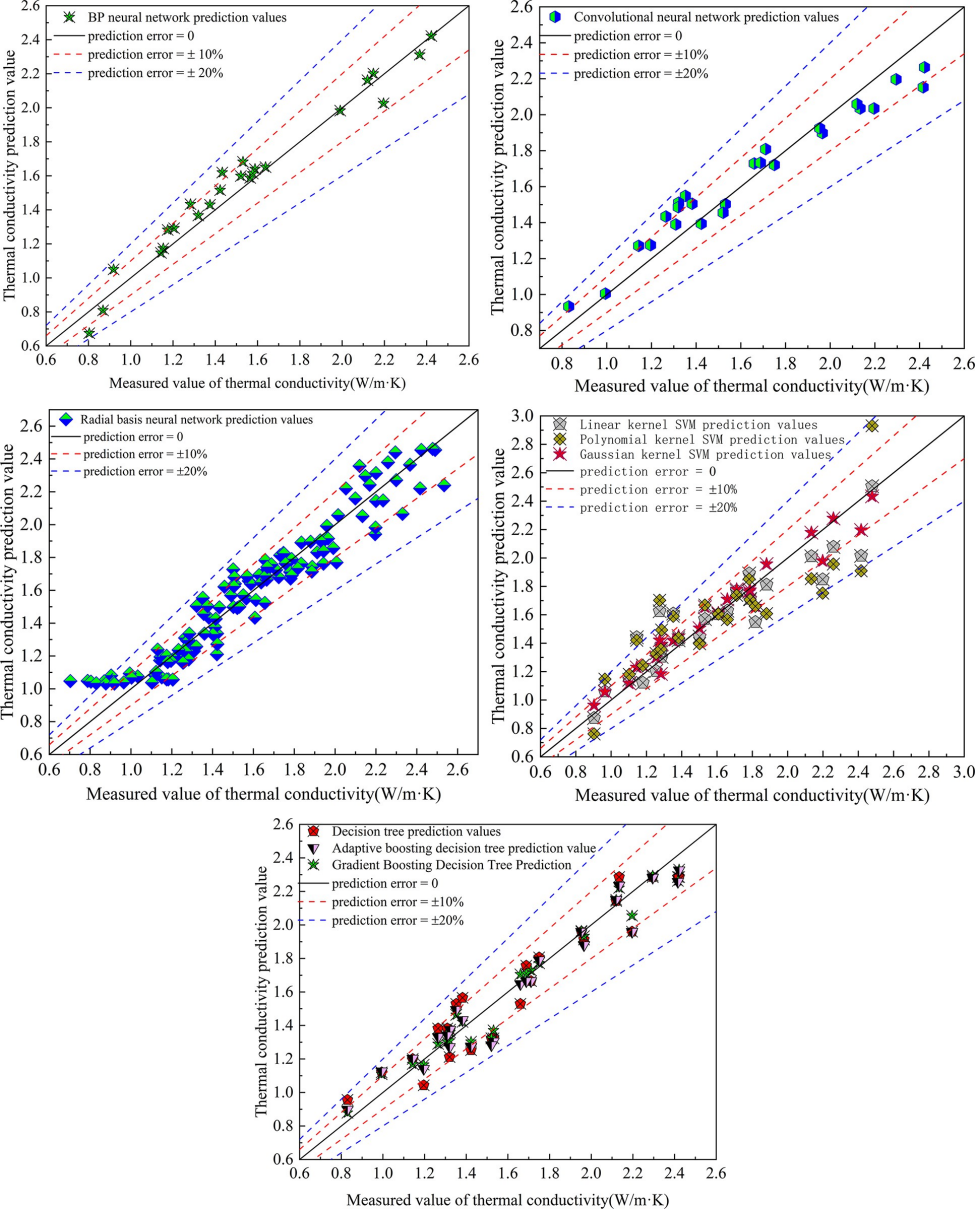
Prediction results of thermal conductivity of different models.

In recent years, intelligent prediction models have been widely used in various fields. Because of its high universality and robustness, it is often used in recognition classification, regression prediction and so on. Based on 125 measured data, models such as BP neural network, convolutional neural network, radial basis neural network, support vector machine neural network and decision tree were established to predict and analyze the evolution law of thermal conductivity of improved soil under the influence of water content, dry density and temperature. As shown in Fig 6, the prediction results of different models on the thermal conductivity of the improved soil are shown.

Firstly, when establishing an intelligent prediction model, we divide the data set into a training set and a test set using the same criteria. The training set is mainly used for model training, and the test set is used to verify the accuracy of the model.

BP neural network consists of three layers: input layer, output layer and hidden layer [23, 24]. In the establishment of BP neural network, the data set is divided into training set and test set, accounting for 80% and 20% respectively. The input layer is set up with three neurons, which are expressed as: moisture content, dry density and temperature; one neuron is set in the output layer to represent the thermal conductivity of frozen soil. Based on the touch method, the hidden layer is set up as a two-layer structure, which contains 5 and 7 neurons respectively. We chose ’ Relu ’ as the activation function and ’ rmsprop ’ [25] as the optimizer. In order to ensure the accuracy and computational efficiency of the model, the number of iterations of model training is set to 1000 times. In order to better show the prediction effect of the model, the BP neural network prediction analysis diagram is drawn with the measured value as the abscissa and the predicted value as the ordinate. As shown in Fig 6(A), the real line in the diagram is the isoline of the measured value and the predicted value. The closer to the real line, the closer the predicted value is to the measured value, the more accurate the predicted data is, and the imaginary line is the error line. From Fig 6(A), it can be seen that the predicted values of BP neural network are less than 20%, and the data with prediction error less than 10% account for 76%. The maximum error is 16.30%, the minimum error is 0, and the mean square error of the predicted value is 0.011.

Convolutional neural network is similar to BP neural network, which belongs to feedforward neural network. Its hidden layer has convolution structure and pooling layer [26]. Based on different application fields, convolutional neural networks can be divided into one-dimensional convolutional neural networks for data regression prediction and two-dimensional convolutional neural networks for image recognition and classification. Based on the prediction of the thermal conductivity of the improved soil, a one-dimensional convolutional neural network with four convolutional layers is established. Each convolutional layer contains a convolution kernel, and a pooling layer is set between each two convolutional layers. The convolutional layer uses ’ Relu ’ as the activation function, the optimizer uses stochastic gradient descent (SGD), and the loss function is MSE.A one-dimensional convolutional neural network with a learning rate of 0.001 and 100 iterations is constructed to predict the thermal conductivity of the soil. The results are shown in Fig 6(B). The results show that 72% of the predicted value error is less than 10%, the maximum prediction error is 14.6%, the minimum prediction error is 1.1%, and the mean square error of the predicted value is 0.014.

Radial basis neural network is a kind of forward neural network with radial symmetry and single hidden layer structure [27]. Based on the need to predict the thermal conductivity of the improved soil in this paper, when establishing the radial basis neural network, three neurons are set in the input layer. According to the test data criterion, 10 neurons are set in the hidden layer, and one neuron is set in the output layer. The 125 data are divided into training set and verification set, of which the training set accounts for 68%, the verification set accounts for 32%, and the test set is all data. The comparison between the predicted results and the measured values is shown in Fig 6(C). The results show that the error of 79.2% thermal conductivity prediction value is less than 10%, the error of 4% thermal conductivity prediction value is more than 20%, the maximum prediction error is 48.9%, the minimum prediction error is 0, and the mean square error of prediction value is 0.014.

Support vector machine is a machine learning algorithm model based on statistical theory [28, 29]. Many scholars have improved the kernel function of support vector machine to improve the universality and accuracy of the model. In this paper, based on the influence of moisture content, dry density and temperature on the thermal conductivity of soil, the support vector machine models with Gaussian kernel, linear kernel and polynomial kernel are established respectively. When adjusting the model parameters, the ’ GridSearchCV ’ is uniformly adopted. At the same time, the data set is divided into training set and test set, accounting for 80% and 20% respectively. The thermal conductivity of lime-modified red clay was predicted by establishing different kernel function support vector machine models, as shown in Fig 6(D). The results show that the predicted values of linear kernel, Gaussian kernel and polynomial kernel support vector machine with prediction error less than 10% account for 76%, 92% and 60% respectively, and the data with prediction error greater than 20% account for 8%, 0 and 16% respectively. The maximum prediction errors are 21.5%, 11.1% and 33.5% respectively, the minimum prediction errors are 0.2%, 0.3% and 0.1% respectively, and the mean square errors of predicted values are 0.029,0.009 and 0.056 respectively.

A decision tree is a machine algorithm model with a ’ tree ’ structure [30]. It is the same as BP neural network and convolutional neural network, and has a three-layer structure. Including: roots, stems, leaves, respectively, representing different treatment stages. Similar to support vector machine, in the application of decision tree, many scholars have also carried out more research on its algorithm. In this study, traditional decision tree, gradient boosting (GBDT) decision tree [31] and adaptive boosting (AdBoost) decision tree [32] were established. Firstly, the data set is divided according to the structure of 80% training set and 20% test set. The maximum depth of the decision tree is set to 4, the number of iterations is 500, and the learning rate is 0.1. The comparison between the predicted value and the measured value of the decision tree of the three different algorithms is shown in Fig 6(E). The results show that the prediction errors of the three decision tree models are less than 10%, accounting for 64%, 88% and 76% respectively. The maximum prediction errors were 15.2%, 12.9% and 15.7%, respectively. The minimum prediction errors are: 0.4%, 0, 0.4%; the mean square errors of the predicted values were 0.017,0.007 and 0.12, respectively.

It can be seen that by establishing five intelligent prediction models, among which the support vector machine and the decision tree model contain a variety of algorithms, the prediction of the thermal conductivity of lime-improved soil under the influence of various factors such as moisture content, dry density and temperature is realized. Among them, among the established support vector machine models with different accounting methods, the support vector machine model with Gaussian kernel has the best effect. Among the established decision tree models with different improved algorithms, the Gradient boosting decision tree has the best prediction effect.

### 4.3 Model evaluation

**Fig 7:**
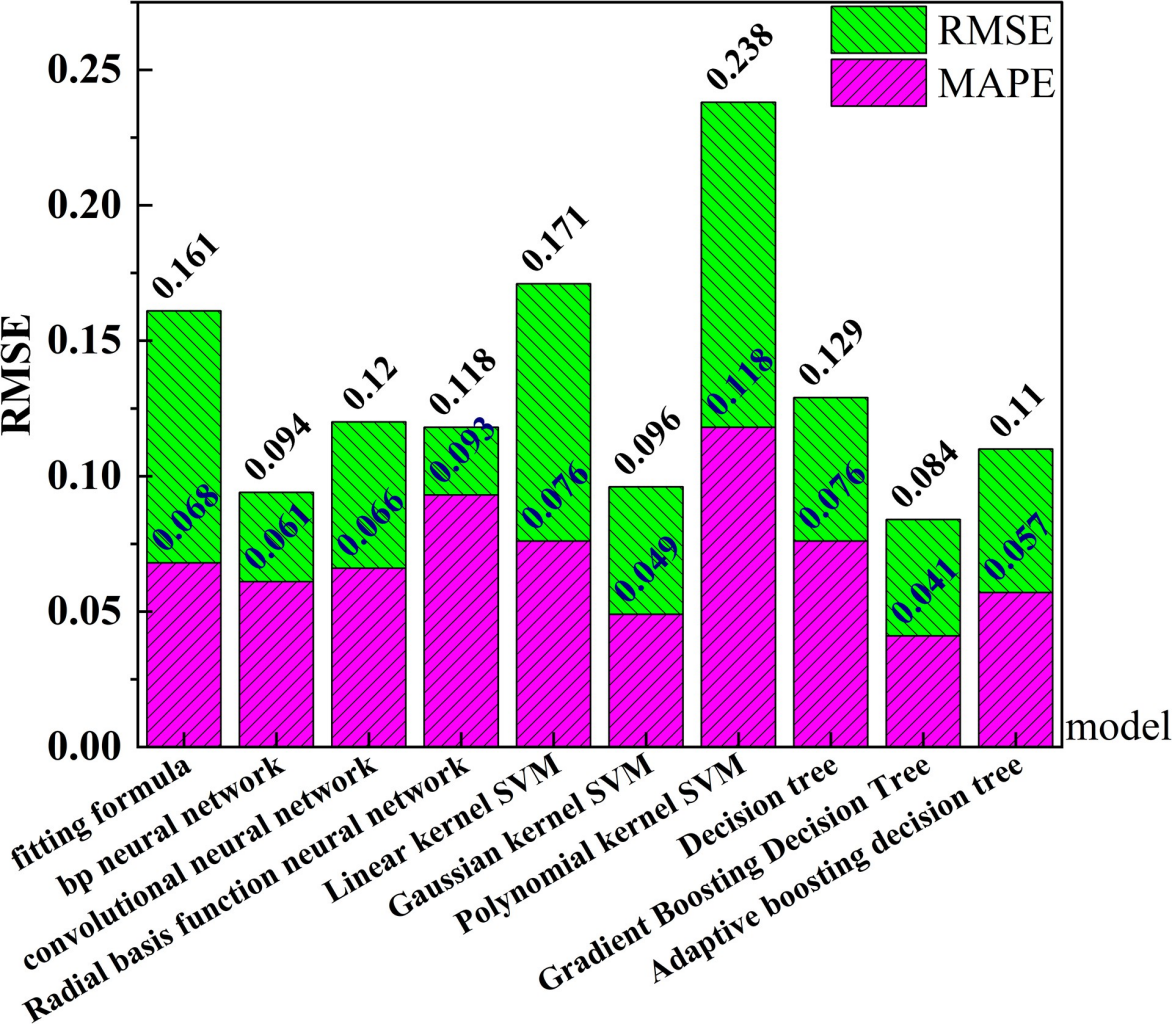
Model evaluation.

The root mean square error (RMSE) and mean absolute percentage error (MAPE) were used to evaluate the above models comprehensively for further implementation of the fitting formula and accurate evaluation of multiple intelligent prediction models. The model evaluation results are shown in Fig 7. The results show that the fitting formula and the intelligent prediction model have achieved good prediction results, and the model progress is high. Gradient boosting decision tree has the best prediction effect, and it is recommended as the prediction model of thermal conductivity of improved soil.

## 5. Conclusion

In this study, the evolution pattern of lime modified red clay under the factors of moisture content, dry density and temperature was investigated. Fitting equations and intelligent prediction models under the influence of various factors were developed. The following conclusions were drawn.

The thermal conductivity of lime modified red clay increased linearly with moisture content and dry density. In the range of 19–27% moisture content, the overall average increase in thermal conductivity was 0.048 W·m^-1^·K^-1^ for each 1% increase in moisture content, and in the range of 1.2–1.6 g·cm^−3^ dry density, the overall average increase in thermal conductivity was 0.197 W·m^-1^·K^-1^ for each 0.1 increase in dry density.

The effect of temperature on the thermal conductivity of lime modified red clay is divided into three stages. In the first stage, in the temperature interval of -2°C to 0°C, the thermal conductivity increased slowly with the decrease of temperature, and the change was small. In the second stage, in the temperature interval of -5°C to (-2°C, the thermal conductivity showed a rapid increase as the temperature decreased. In the third stage, in the -10°C to (-5°C interval, the thermal conductivity changes less with decreasing temperature, and the fitted curves approximate to be smooth.

By establishing the fitted model and intelligent prediction model and comparing them with the previous empirical model, it was found that the previous empirical model was affected by the soil properties and other factors, and its universality was low, and the prediction effect was poor; the established fitted model and intelligent prediction model both achieved good prediction of the thermal conductivity of lime modified red clay at low temperature, and the prediction accuracy was high.

The intelligent prediction models generally outperformed the fitted models, with higher accuracy and greater generalization. The GBDT decision tree model is preferred for the prediction of the thermal conductivity of the improved soil.

The thermal conductivity of soil is not only affected by factors such as soil moisture content, dry density and temperature, but also by factors such as mineral composition and physical properties of soil. Therefore, at present, both theoretical models and empirical models are not universal. It may be more suitable for similar soils, and it is not completely universal. When applied to the prediction of thermal conductivity of other soils, the corresponding model correction is needed.

## Supporting information

S1 Table Measured data. (DOCX)
