# Supplementary material for: Study on thermal conductivity of improved soil under different freezing temperatures
Source: PLoS One. 2023 Oct 18;18(10):e0292560. doi: 10.1371/journal.pone.0292560 (PMC10584184; doi:10.1371/journal.pone.0292560)
Supplement: S1 Table — (DOCX) [file pone.0292560.s001.docx]

**S1 Table Measured data**

| moisture content  /(%) | Dry density  /(g·cm^−3^) | Temperature  /(℃) | Thermal conductivity.  /(W·m^-1^·K^-1^) |
| --- | --- | --- | --- |
| 19 | 1.2 | 0 | 0.703 |
| 19 | 1.2 | -3 | 0.903 |
| 19 | 1.2 | -5 | 1.143 |
| 19 | 1.2 | -7 | 1.154 |
| 19 | 1.2 | -10 | 1.145 |
| 19 | 1.3 | 0 | 0.788 |
| 19 | 1.3 | -3 | 1.033 |
| 19 | 1.3 | -5 | 1.309 |
| 19 | 1.3 | -7 | 1.265 |
| 19 | 1.3 | -10 | 1.275 |
| 19 | 1.4 | 0 | 0.994 |
| 19 | 1.4 | -3 | 1.222 |
| 19 | 1.4 | -5 | 1.565 |
| 19 | 1.4 | -7 | 1.577 |
| 19 | 1.4 | -10 | 1.588 |
| 19 | 1.5 | 0 | 1.131 |
| 19 | 1.5 | -3 | 1.382 |
| 19 | 1.5 | -5 | 1.731 |
| 19 | 1.5 | -7 | 1.747 |
| 19 | 1.5 | -10 | 1.834 |
| 19 | 1.6 | 0 | 1.272 |
| 19 | 1.6 | -3 | 1.607 |
| 19 | 1.6 | -5 | 1.953 |
| 19 | 1.6 | -7 | 1.960 |
| 19 | 1.6 | -10 | 2.196 |
| 21 | 1.2 | 0 | 0.804 |
| 21 | 1.2 | -3 | 1.003 |
| 21 | 1.2 | -5 | 1.260 |
| 21 | 1.2 | -7 | 1.175 |
| 21 | 1.2 | -10 | 1.267 |
| 21 | 1.3 | 0 | 0.869 |
| 21 | 1.3 | -3 | 1.151 |
| 21 | 1.3 | -5 | 1.421 |
| 21 | 1.3 | -7 | 1.422 |
| 21 | 1.3 | -10 | 1.412 |
| 21 | 1.4 | 0 | 1.120 |
| 21 | 1.4 | -3 | 1.283 |
| 21 | 1.4 | -5 | 1.612 |
| 21 | 1.4 | -7 | 1.628 |
| 21 | 1.4 | -10 | 1.782 |
| 21 | 1.5 | 0 | 1.233 |
| 21 | 1.5 | -3 | 1.433 |
| 21 | 1.5 | -5 | 1.835 |
| 21 | 1.5 | -7 | 1.965 |
| 21 | 1.5 | -10 | 1.991 |
| 21 | 1.6 | 0 | 1.320 |
| 21 | 1.6 | -3 | 1.656 |
| 21 | 1.6 | -5 | 2.198 |
| 21 | 1.6 | -7 | 2.134 |
| 21 | 1.6 | -10 | 2.330 |
| 23 | 1.2 | 0 | 0.830 |
| 23 | 1.2 | -3 | 1.123 |
| 23 | 1.2 | -5 | 1.402 |
| 23 | 1.2 | -7 | 1.415 |
| 23 | 1.2 | -10 | 1.352 |
| 23 | 1.3 | 0 | 0.916 |
| 23 | 1.3 | -3 | 1.196 |
| 23 | 1.3 | -5 | 1.502 |
| 23 | 1.3 | -7 | 1.509 |
| 23 | 1.3 | -10 | 1.638 |
| 23 | 1.4 | 0 | 1.183 |
| 23 | 1.4 | -3 | 1.423 |
| 23 | 1.4 | -5 | 1.724 |
| 23 | 1.4 | -7 | 1.750 |
| 23 | 1.4 | -10 | 1.888 |
| 23 | 1.5 | 0 | 1.282 |
| 23 | 1.5 | -3 | 1.665 |
| 23 | 1.5 | -5 | 2.015 |
| 23 | 1.5 | -7 | 2.200 |
| 23 | 1.5 | -10 | 2.099 |
| 23 | 1.6 | 0 | 1.357 |
| 23 | 1.6 | -3 | 1.711 |
| 23 | 1.6 | -5 | 2.235 |
| 23 | 1.6 | -7 | 2.299 |
| 23 | 1.6 | -10 | 2.367 |
| 25 | 1.2 | 0 | 0.877 |
| 25 | 1.2 | -3 | 1.180 |
| 25 | 1.2 | -5 | 1.491 |
| 25 | 1.2 | -7 | 1.501 |
| 25 | 1.2 | -10 | 1.507 |
| 25 | 1.3 | 0 | 0.965 |
| 25 | 1.3 | -3 | 1.286 |
| 25 | 1.3 | -5 | 1.669 |
| 25 | 1.3 | -7 | 1.658 |
| 25 | 1.3 | -10 | 1.660 |
| 25 | 1.4 | 0 | 1.175 |
| 25 | 1.4 | -3 | 1.521 |
| 25 | 1.4 | -5 | 1.912 |
| 25 | 1.4 | -7 | 1.880 |
| 25 | 1.4 | -10 | 1.943 |
| 25 | 1.5 | 0 | 1.322 |
| 25 | 1.5 | -3 | 1.688 |
| 25 | 1.5 | -5 | 2.053 |
| 25 | 1.5 | -7 | 2.120 |
| 25 | 1.5 | -10 | 2.148 |
| 25 | 1.6 | 0 | 1.458 |
| 25 | 1.6 | -3 | 1.780 |
| 25 | 1.6 | -5 | 2.416 |
| 25 | 1.6 | -7 | 2.295 |
| 25 | 1.6 | -10 | 2.422 |
| 27 | 1.2 | 0 | 0.919 |
| 27 | 1.2 | -3 | 1.255 |
| 27 | 1.2 | -5 | 1.550 |
| 27 | 1.2 | -7 | 1.668 |
| 27 | 1.2 | -10 | 1.569 |
| 27 | 1.3 | 0 | 1.103 |
| 27 | 1.3 | -3 | 1.360 |
| 27 | 1.3 | -5 | 1.817 |
| 27 | 1.3 | -7 | 1.787 |
| 27 | 1.3 | -10 | 1.787 |
| 27 | 1.4 | 0 | 1.205 |
| 27 | 1.4 | -3 | 1.531 |
| 27 | 1.4 | -5 | 1.944 |
| 27 | 1.4 | -7 | 1.927 |
| 27 | 1.4 | -10 | 2.007 |
| 27 | 1.5 | 0 | 1.375 |
| 27 | 1.5 | -3 | 1.757 |
| 27 | 1.5 | -5 | 2.169 |
| 27 | 1.5 | -7 | 2.259 |
| 27 | 1.5 | -10 | 2.200 |
| 27 | 1.6 | 0 | 1.505 |
| 27 | 1.6 | -3 | 1.886 |
| 27 | 1.6 | -5 | 2.534 |
| 27 | 1.6 | -7 | 2.490 |
| 27 | 1.6 | -10 | 2.478 |
